# Supplementary material for: Primary exposure to Zika virus increases risk of symptomatic dengue virus infection with serotypes 2, 3, and 4 but not serotype 1
Source: medRxiv. 2023 Nov 30:2023.11.29.23299187. Preprint. [Version 1] doi: 10.1101/2023.11.29.23299187 (PMC10705633; doi:10.1101/2023.11.29.23299187)
Supplement: Supplement 1 [file media-1.pdf]

SUPPLEMENTARY MATERIAL

Primary exposure to Zika virus increases risk of symptomatic dengue virus infection with serotypes 2, 3, and 4 but not serotype 1

Jose Victor Zambrana[1,2]†, Chloe M. Hasund[3]†, Rosemary A. Aogo[3]†, Sandra Bos[4], Sonia Arguello[1], Karla Gonzalez[1,5], Damaris Collado[1], Tatiana Miranda[1], Guillermina Kuan[1,6], Aubree Gordon[2], Angel Balmaseda[1,5], Leah Katzelnick[3*], Eva Harris[4*]

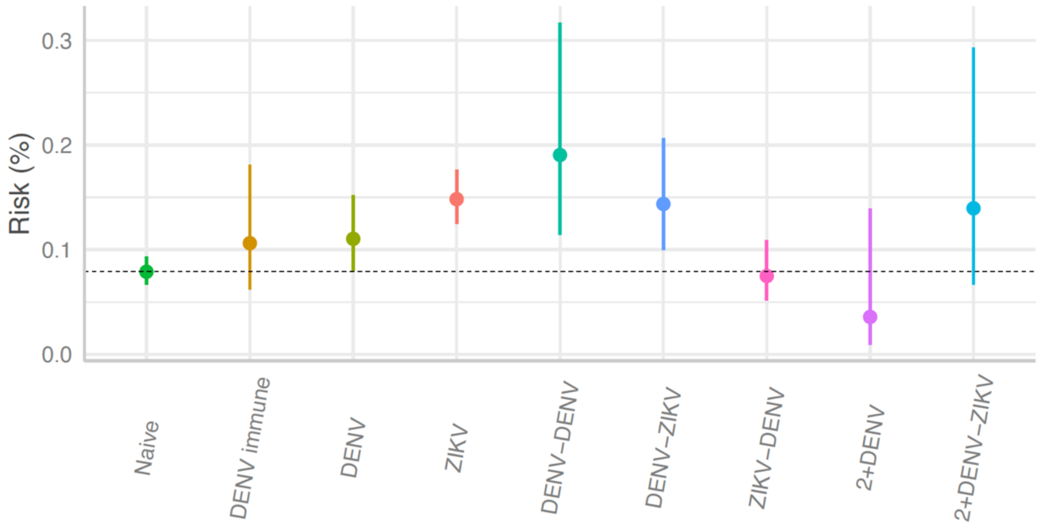

Fig. S1. Risk of dengue differs by infection history on crude levels. Crude risk of symptomatic DENV infection by prior DENV and ZIKV infection histories, 2022-2023.

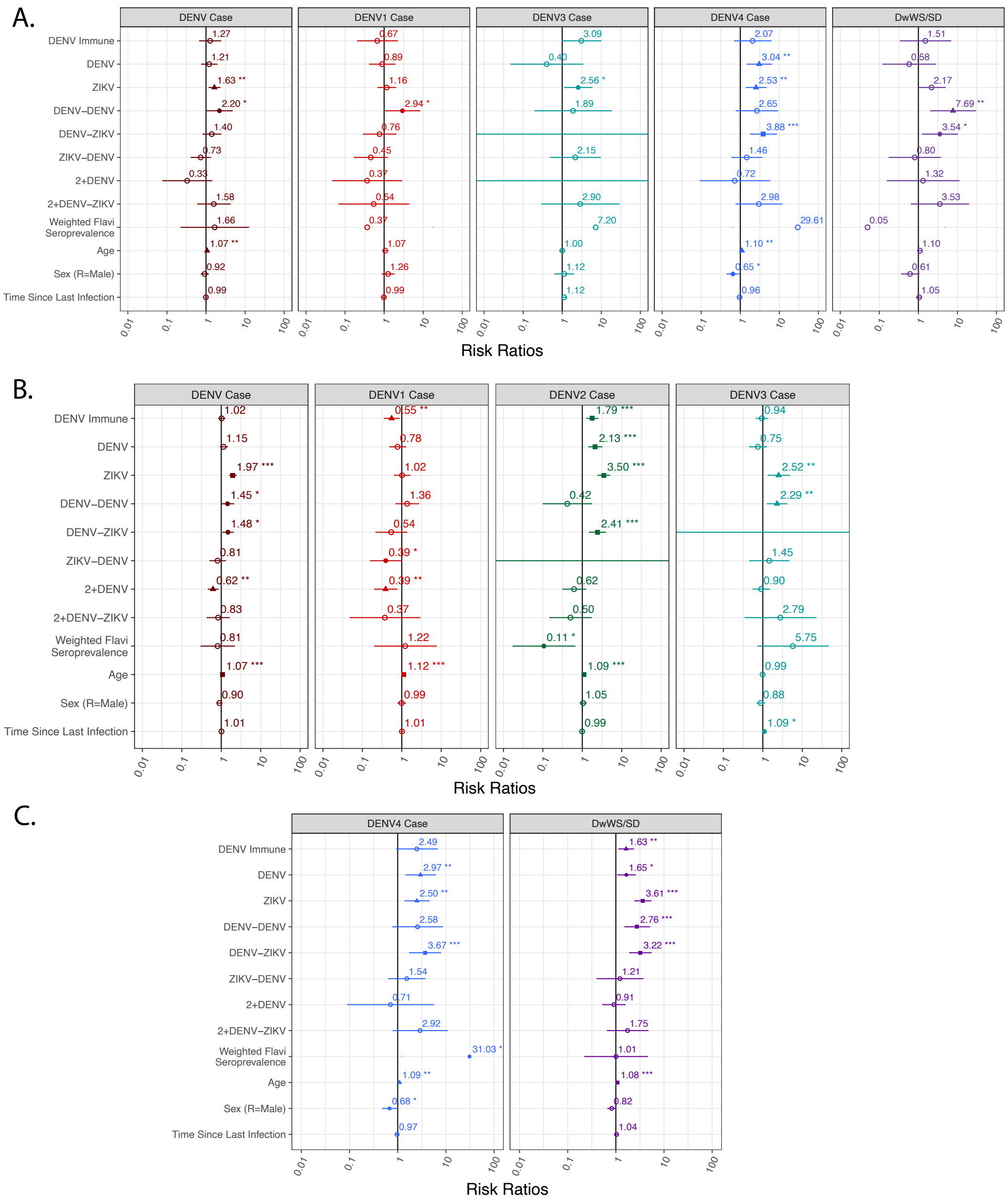

Fig. S2. Prior infection history confers differential risk of symptomatic DENV1-4 considering baseline spatial risk. Risks ratios of symptomatic and severe dengue by prior DENV and ZIKV infection histories including age-adjusted flavivirus seroprevalence of the prior year on the neighborhood level as a covariate **(A)** in the 2022-2023 epidemic season **(B-C)** in the entire cohort season (2004-2023).

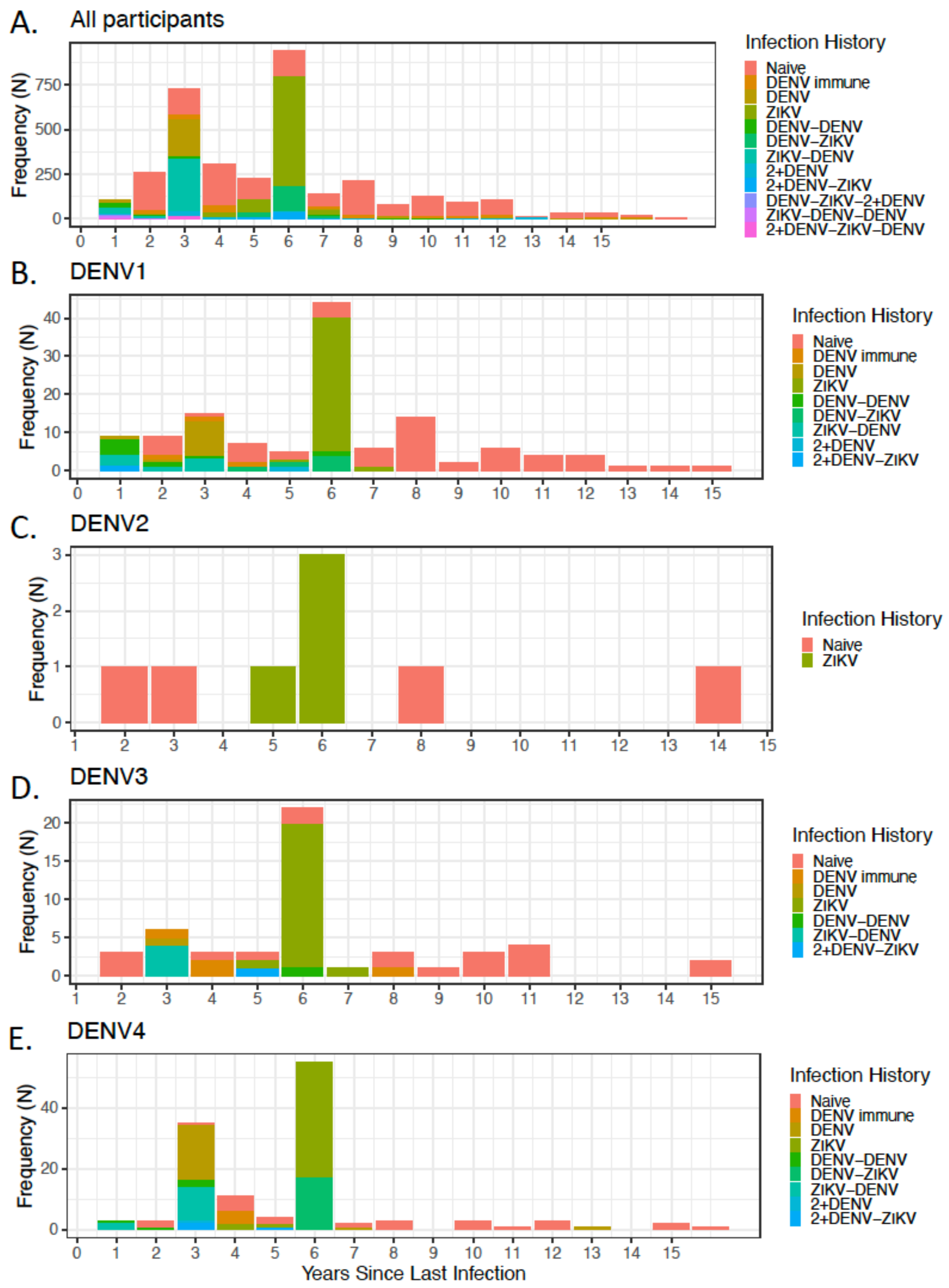

Fig. S3. Most secondary infections in 2022 had DENV2 and/or ZIKV infections as a primary infection. Frequency of DENV cases **(A)** and cases due to DENV1 **(B)**, DENV2 **(C)**, DENV3 **(D)**, and DENV4 **(E)** infections in 2022 by years since last infection for each infection history. The flavivirus-naïve group contains years since entered the cohort.

Table S1. Characteristics of participants in the Pediatric Dengue Cohort Study with documented infection histories, 2004-2023

Variable	DENV Cases						
	**Overall,** N = 1,418[1]	**DENV1,** N = 294[1]	**DENV2,** N = 481[1]	**DENV3,** N = 310[1]	**DENV4,** N = 135[1]	**Dengue Serology,** N = 178[1]	**P-value** [2]
**Age**	9.5 (6.8, 12.2)	9.9 (7.4, 12.5)	9.5 (7.0, 12.1)	8.8 (6.0, 11.1)	11.9 (9.8, 14.3)	8.2 (5.7, 10.7)	<0.001
**Sex**							0.2
F	738 (53%)	149 (51%)	239 (50%)	159 (52%)	78 (58%)	113 (58%)	
M	667 (47%)	142 (49%)	239 (50%)	148 (48%)	56 (42%)	82 (42%)	
**DHF/DSS**	66 (4.7%)	8 (2.7%)	30 (6.2%)	28 (9.0%)	0 (0%)	0 (0%)	<0.001
**DwWS/SD**	383 (27%)	62 (21%)	182 (38%)	103 (33%)	24 (18%)	12 (6.1%)	<0.001
**Time Since Last Infection**	4.54 (3.00, 6.00)	5.36 (3.00, 8.00)	3.72 (3.00, 4.00)	4.70 (3.00, 6.00)	5.35 (3.00, 6.00)	4.58 (2.00, 7.00)	<0.001
**Geometric Mean Titer (iELISA)**	384 (2, 126)	214 (2, 43)	514 (2, 197)	141 (2, 64)	675 (2, 289)	491 (2, 39)	<0.001
**Infection History**							
Naive	523 (38%)	139 (49%)	115 (24%)	134 (45%)	24 (19%)	111 (58%)	
DENV immune	215 (16%)	36 (13%)	86 (18%)	67 (23%)	6 (4.7%)	20 (10%)	
DENV	124 (9.0%)	29 (10%)	39 (8.2%)	21 (7.1%)	20 (16%)	15 (7.9%)	
ZIKV	306 (22%)	39 (14%)	180 (38%)	21 (7.1%)	41 (32%)	25 (13%)	
DENV-DENV	41 (3.0%)	13 (4.6%)	2 (0.4%)	18 (6.1%)	4 (3.1%)	4 (2.1%)	
DENV-ZIKV	71 (5.2%)	6 (2.1%)	43 (9.0%)	0 (0%)	17 (13%)	5 (2.6%)	
ZIKV-DENV	26 (1.9%)	7 (2.5%)	0 (0%)	4 (1.3%)	13 (10%)	2 (1.0%)	
2+DENV	60 (4.4%)	12 (4.3%)	10 (2.1%)	31 (10%)	1 (0.8%)	6 (3.1%)	
2+DENV-ZIKV	11 (0.8%)	1 (0.4%)	3 (0.6%)	1 (0.3%)	3 (2.3%)	3 (1.6%)	
DENV-ZIKV-2+DENV	0 (0%)	0 (0%)	0 (0%)	0 (0%)	0 (0%)	0 (0%)	
ZIKV-DENV-DENV	0 (0%)	0 (0%)	0 (0%)	0 (0%)	0 (0%)	0 (0%)	
2+DENV-ZIKV-DENV	0 (0%)	0 (0%)	0 (0%)	0 (0%)	0 (0%)	0 (0%)	

[1]Mean (IQR); n (%)
[2]Kruskal-Wallis rank sum test; Pearson's Chi-squared test

Table S2. Estimated risk ratios of a dengue case by prior DENV and ZIKV infection histories, 2022-2023.

	**DENV Case**			**DENV1 Case**			**DENV3 Case**			**DENV4 Case**			**DwWS/SD**		
Predictors	*Risk Ratios*	*CI*	*p*	*Risk Ratios*	*CI*	*p*	*Risk Ratios*	*CI*	*p*	*Risk Ratios*	*CI*	*p*	*Incidence Rate Ratios*	*CI*	*p*
(Intercept)	0.06	0.04 – 0.08	**<0.001**	0.02	0.01 – 0.04	**<0.001**	0.01	0.00 – 0.02	**<0.001**	0.01	0.01 – 0.03	**<0.001**	0.00	0.00 – 0.01	**<0.001**
inf hist DD [DENV immune]	1.26	0.72 – 2.23	0.420	0.69	0.21 – 2.18	0.523	3.23	1.09 – 9.57	**0.034**	2.08	0.73 – 5.96	0.171	1.60	0.37 – 7.04	0.532
inf hist DD [DENV]	1.25	0.82 – 1.89	0.296	0.91	0.44 – 1.88	0.801	0.38	0.05 – 3.00	0.359	3.25	1.65 – 6.41	**0.001**	0.60	0.13 – 2.81	0.515
inf hist DD [ZIKV]	1.61	1.20 – 2.17	**0.002**	1.20	0.72 – 1.99	0.479	2.90	1.34 – 6.27	**0.007**	2.62	1.48 – 4.63	**0.001**	2.19	0.99 – 4.83	0.053
inf hist DD [DENV-DENV]	1.96	1.05 – 3.66	**0.036**	2.45	0.97 – 6.20	0.058	2.20	0.25 – 19.20	0.476	2.58	0.81 – 8.26	0.109	6.79	1.88 – 24.58	**0.003**
inf hist DD [DENV-ZIKV]	1.43	0.90 – 2.29	0.131	0.76	0.30 – 1.93	0.567	0.00	0.00 – Inf	0.984	3.96	1.93 – 8.12	**<0.001**	3.46	1.24 – 9.65	**0.018**
inf hist DD [ZIKV-DENV]	0.79	0.47 – 1.34	0.384	0.48	0.19 – 1.24	0.130	1.76	0.43 – 7.13	0.431	1.60	0.68 – 3.76	0.282	0.82	0.18 – 3.69	0.797
inf hist DD [2+DENV]	0.37	0.09 – 1.50	0.164	0.39	0.05 – 2.94	0.364	0.00	0.00 – Inf	0.991	0.78	0.10 – 5.98	0.815	1.37	0.17 – 11.06	0.768
inf hist DD [2+DENV-ZIKV]	1.28	0.56 – 2.92	0.563	0.46	0.06 – 3.50	0.454	2.62	0.30 – 23.26	0.387	2.56	0.72 – 9.06	0.146	2.85	0.54 – 15.01	0.217
inf.hist.DDDENV-ZIKV-2+DENV	0.00	0.00 – Inf	0.986	0.00	0.00 – Inf	0.992	0.00	0.00 – Inf	0.998	0.00	0.00 – Inf	0.992	0.00	0.00 – Inf	0.995
age	1.04	1.00 – 1.08	**0.029**	1.05	0.98 – 1.12	0.150	0.98	0.88 – 1.09	0.687	1.07	1.01 – 1.14	**0.023**	1.07	0.98 – 1.18	0.141
sex [M]	0.94	0.77 – 1.14	0.525	1.26	0.89 – 1.77	0.193	1.14	0.66 – 1.97	0.634	0.67	0.47 – 0.95	**0.025**	0.62	0.37 – 1.04	0.069
time per imm class	1.00	0.95 – 1.05	0.910	0.99	0.91 – 1.08	0.841	1.12	0.98 – 1.29	0.100	0.96	0.88 – 1.05	0.391	1.06	0.94 – 1.20	0.369
Observations	3411			3411			3411			3411			3411		
R^2 Nagelkerke	0.033			0.022			0.053			0.071			0.087		

Table S3. Estimated risk ratios of a dengue case by prior DENV and ZIKV infection histories, 2004-2023

	DENV Case			DENV1 Case			DENV2 Case			DENV3 Case			DENV4 Case			DwWS/SD		
Predictors	*Incidence Rate Ratios*	*CI*	*p*	*Risk Ratios*	*CI*	*p*	*Risk Ratios*	*CI*	*p*	*Incidence Rate Ratios*	*CI*	*p*	*Incidence Rate Ratios*	*CI*	*p*	*Incidence Rate Ratios*	*CI*	*p*
(Intercept)	0.00	0.00 – 0.01	**<0.001**	0.00	0.00 – 0.00	**<0.001**	0.00	0.00 – 0.00	**<0.001**	0.00	0.00 – Inf	0.996	0.00	0.00 – 0.00	**<0.001**	0.00	0.00 – 0.00	**<0.001**
inf hist DD [DENV immune]	1.04	0.87 – 1.25	0.649	0.57	0.38 – 0.86	**0.008**	1.84	1.33 – 2.55	**<0.001**	0.99	0.71 – 1.40	0.973	2.65	1.03 – 6.83	**0.044**	1.67	1.16 – 2.39	**0.006**
inf hist DD [DENV]	1.17	0.94 – 1.45	0.160	0.80	0.51 – 1.27	0.346	2.09	1.44 – 3.05	**<0.001**	0.78	0.48 – 1.29	0.341	3.28	1.64 – 6.57	**0.001**	1.67	1.09 – 2.57	**0.019**
inf hist DD [ZIKV]	1.87	1.54 – 2.27	**<0.001**	1.04	0.66 – 1.63	0.856	3.62	2.60 – 5.04	**<0.001**	2.60	1.39 – 4.85	**0.003**	2.66	1.49 – 4.76	**0.001**	3.62	2.44 – 5.38	**<0.001**
inf hist DD [DENV-DENV]	1.45	1.03 – 2.04	**0.035**	1.37	0.72 – 2.61	0.335	0.37	0.09 – 1.51	0.164	2.40	1.37 – 4.23	**0.002**	2.64	0.80 – 8.68	0.110	2.78	1.53 – 5.03	**0.001**
inf hist DD [DENV-ZIKV]	1.47	1.09 – 1.99	**0.012**	0.57	0.24 – 1.37	0.210	2.53	1.64 – 3.91	**<0.001**	0.00	0.00 – Inf	0.997	4.05	1.93 – 8.49	**<0.001**	3.26	1.94 – 5.50	**<0.001**
inf hist DD [ZIKV-DENV]	0.85	0.55 – 1.34	0.490	0.42	0.18 – 1.02	0.055	0.00	0.00 – Inf	0.990	1.47	0.47 – 4.66	0.510	1.67	0.70 – 4.00	0.249	1.22	0.41 – 3.67	0.724
inf hist DD [2+DENV]	0.64	0.48 – 0.86	**0.003**	0.41	0.22 – 0.79	**0.008**	0.55	0.28 – 1.09	0.086	0.96	0.60 – 1.56	0.883	0.76	0.10 – 5.86	0.790	0.93	0.54 – 1.60	0.785
inf hist DD [2+DENV-ZIKV]	0.82	0.44 – 1.54	0.533	0.32	0.04 – 2.36	0.263	0.45	0.14 – 1.46	0.183	2.33	0.30 – 18.05	0.418	2.60	0.71 – 9.56	0.151	1.72	0.64 – 4.58	0.280
inf.hist.DDDENV-ZIKV-2+DENV	0.00	0.00 – Inf	0.999	0.00	0.00 – Inf	1.000	0.00	0.00 – Inf	1.000	0.00	0.00 – Inf	1.000	0.00	0.00 – Inf	1.000	0.00	0.00 – Inf	1.000
age	1.05	1.03 – 1.08	**<0.001**	1.10	1.05 – 1.16	**<0.001**	1.09	1.05 – 1.12	**<0.001**	0.98	0.93 – 1.04	0.542	1.08	1.01 – 1.14	**0.020**	1.08	1.04 – 1.12	**<0.001**
sex [M]	0.90	0.81 – 1.00	0.052	0.97	0.77 – 1.23	0.827	1.04	0.88 – 1.23	0.666	0.88	0.70 – 1.11	0.288	0.69	0.49 – 0.99	**0.042**	0.83	0.68 – 1.01	0.065
time per imm class	1.01	0.98 – 1.04	0.421	1.01	0.96 – 1.08	0.627	1.00	0.95 – 1.06	0.968	1.09	1.01 – 1.18	**0.020**	0.97	0.88 – 1.06	0.482	1.04	0.98 – 1.10	0.242
cohort year [2005]	3.53	2.01 – 6.22	**<0.001**	2.58	1.16 – 5.73	**0.020**	8.03	2.44 – 26.41	**0.001**	0.94	0.00 – Inf	1.000	0.00	0.00 – Inf	1.000	1.70	0.15 – 18.78	0.664
cohort year [2006]	0.73	0.34 – 1.53	0.399	0.09	0.01 – 0.76	**0.027**	2.61	0.70 – 9.64	0.151	0.90	0.00 – Inf	1.000	0.00	0.00 – Inf	1.000	0.80	0.05 – 12.79	0.873
cohort year [2007]	3.58	2.03 – 6.32	**<0.001**	0.09	0.01 – 0.74	**0.025**	19.11	5.96 – 61.28	**<0.001**	0.86	0.00 – Inf	1.000	0.00	0.00 – Inf	1.000	12.26	1.61 – 93.44	**0.016**
cohort year [2008]	1.12	0.58 – 2.18	0.729	0.00	0.00 – Inf	1.000	0.56	0.09 – 3.40	0.532	378254773.16	0.00 – Inf	0.997	0.00	0.00 – Inf	1.000	3.62	0.42 – 31.28	0.242
cohort year [2009]	7.80	4.54 – 13.39	**<0.001**	1.18	0.49 – 2.83	0.711	2.54	0.68 – 9.52	0.166	2448918720.52	0.00 – Inf	0.997	0.00	0.00 – Inf	1.000	46.10	6.31 – 336.96	**<0.001**
cohort year [2010]	4.67	2.67 – 8.16	**<0.001**	0.00	0.00 – Inf	1.000	4.61	1.32 – 16.06	**0.016**	1320414757.55	0.00 – Inf	0.997	0.00	0.00 – Inf	1.000	18.10	2.42 – 135.68	**0.005**
cohort year [2011]	1.46	0.76 – 2.79	0.255	0.31	0.09 – 1.06	0.063	0.00	0.00 – Inf	0.985	361967558.36	0.00 – Inf	0.997	0.00	0.00 – Inf	1.000	3.83	0.44 – 33.50	0.225
cohort year [2012]	4.28	2.43 – 7.54	**<0.001**	5.05	2.31 – 11.04	**<0.001**	0.28	0.03 – 2.73	0.273	37368050.46	0.00 – Inf	0.997	0.00	0.00 – Inf	1.000	14.08	1.84 – 107.44	**0.011**
cohort year [2013]	1.75	0.94 – 3.27	0.080	1.28	0.53 – 3.08	0.582	1.79	0.43 – 7.34	0.421	77476627.39	0.00 – Inf	0.997	1.04	0.06 – 18.11	0.979	8.53	1.08 – 67.53	**0.042**
cohort year [2014]	0.58	0.26 – 1.28	0.174	0.00	0.00 – Inf	1.000	0.30	0.03 – 2.99	0.307	58115982.30	0.00 – Inf	0.997	0.00	0.00 – Inf	1.000	0.00	0.00 – Inf	0.999
cohort year [2015]	1.51	0.80 – 2.86	0.202	0.00	0.00 – Inf	1.000	7.23	2.11 – 24.78	**0.002**	0.69	0.00 – Inf	1.000	0.00	0.00 – Inf	1.000	3.48	0.39 – 30.74	0.262
cohort year [2016]	1.60	0.85 – 2.99	0.142	0.00	0.00 – Inf	0.999	2.48	0.65 – 9.45	0.184	0.69	0.00 – Inf	1.000	0.00	0.00 – Inf	1.000	0.00	0.00 – Inf	1.000
cohort year [2017]	0.15	0.05 – 0.44	**0.001**	0.00	0.00 – Inf	0.999	0.00	0.00 – Inf	0.983	0.61	0.00 – Inf	1.000	0.00	0.00 – Inf	1.000	0.00	0.00 – Inf	0.999
cohort year [2018]	0.36	0.16 – 0.81	**0.014**	0.00	0.00 – Inf	0.999	0.44	0.09 – 2.26	0.325	0.57	0.00 – Inf	1.000	0.00	0.00 – Inf	1.000	0.00	0.00 – Inf	0.999
cohort year [2019]	11.52	6.67 – 19.89	**<0.001**	0.00	0.00 – Inf	1.000	48.67	14.75 – 160.55	**<0.001**	0.55	0.00 – Inf	1.000	0.00	0.00 – Inf	1.000	56.26	7.54 – 419.97	**<0.001**
cohort year [2020]	0.19	0.07 – 0.53	**0.002**	0.00	0.00 – Inf	0.999	0.38	0.06 – 2.35	0.297	0.58	0.00 – Inf	1.000	0.00	0.00 – Inf	1.000	0.00	0.00 – Inf	0.999
cohort year [2021]	0.12	0.03 – 0.41	**0.001**	0.10	0.02 – 0.50	**0.005**	0.00	0.00 – Inf	0.983	0.56	0.00 – Inf	1.000	0.00	0.00 – Inf	1.000	0.00	0.00 – Inf	1.000
cohort year [2022]	13.06	7.49 – 22.77	**<0.001**	6.76	3.00 – 15.27	**<0.001**	1.60	0.40 – 6.42	0.508	705881112.68	0.00 – Inf	0.997	97.75	11.66 – 819.56	**<0.001**	24.68	3.22 – 189.12	**0.002**
**Random Effects**																		
σ^2	5.05			NA			NA			6.76			7.59			0.00		
τ_{00}	0.00 $_{case.ids}$			0.00 $_{case.ids}$			1.00 $_{case.ids}$			0.00 $_{case.ids}$			0.00 $_{case.ids}$			0.00 $_{case.ids}$		
ICC	0.00												0.00			1.00		
N	9165 $_{case.ids}$			9165 $_{case.ids}$			9165 $_{case.ids}$			9165 $_{case.ids}$			9165 $_{case.ids}$			9165 $_{case.ids}$		
Observations	66842			67438			67438			67438			67438			67438		
Marginal R^2 / Conditional R^2	0.303 / 0.303			NA			NA			0.943 / NA			0.909 / 0.909			1.000 / 1.000		

Table S4. Estimated risk of a dengue case by pre-existing flavivirus immunity, 2004-2023.

	DENV Case			DENV1 Case			DENV2 Case			DENV3 Case			DENV4 Case			DwWS/SD			DHF/DSS		
Predictors	*Risk Ratios*	*CI*	*p*	*Risk Ratios*	*CI*	*p*	*Risk Ratios*	*CI*	*p*	*Risk Ratios*	*CI*	*p*	*Incidence Rate Ratios*	*CI*	*p*	*Risk Ratios*	*CI*	*p*	*Risk Ratios*	*CI*	*p*
(Intercept)	0.00	0.00 – 0.01	**<0.001**	0.00	0.00 – 0.00	**<0.001**	0.00	0.00 – 0.00	**<0.001**	0.00	0.00 – Inf	0.994	0.00	0.00 – 0.00	**<0.001**	0.00	0.00 – 0.00	**<0.001**	0.00	0.00 – Inf	0.994
naive TF [FALSE]	1.28	1.12 – 1.47	**<0.001**	0.72	0.53 – 0.98	**0.036**	2.25	1.75 – 2.90	**<0.001**	1.09	0.82 – 1.46	0.551	2.96	1.73 – 5.09	**<0.001**	2.16	1.63 – 2.87	**<0.001**	4.57	2.06 – 10.12	**<0.001**
age	1.04	1.02 – 1.06	**<0.001**	1.09	1.04 – 1.15	**<0.001**	1.06	1.03 – 1.09	**<0.001**	0.99	0.94 – 1.04	0.644	1.06	1.00 – 1.12	**0.038**	1.07	1.03 – 1.11	**<0.001**	1.04	0.94 – 1.14	0.432
sex [M]	0.89	0.80 – 0.99	**0.026**	0.96	0.76 – 1.21	0.749	1.00	0.84 – 1.19	0.964	0.88	0.71 – 1.11	0.287	0.68	0.48 – 0.97	**0.033**	0.81	0.67 – 0.99	**0.040**	1.13	0.69 – 1.87	0.620
time per imm class	1.01	0.99 – 1.04	0.271	1.02	0.97 – 1.07	0.479	0.99	0.94 – 1.03	0.598	1.08	1.01 – 1.15	**0.023**	1.01	0.93 – 1.08	0.877	1.02	0.97 – 1.07	0.441	1.04	0.92 – 1.19	0.517
cohort year [2005]	3.50	1.99 – 6.15	**<0.001**	2.59	1.17 – 5.74	**0.019**	7.86	2.39 – 25.82	**0.001**	0.01	0.00 – Inf	1.000	0.00	0.00 – Inf	1.000	1.69	0.15 – 18.66	0.667	0.95	0.00 – Inf	1.000
cohort year [2006]	0.71	0.34 – 1.49	0.363	0.10	0.01 – 0.76	**0.027**	2.48	0.67 – 9.16	0.172	0.01	0.00 – Inf	1.000	0.00	0.00 – Inf	1.000	0.78	0.05 – 12.52	0.863	9039689.63	0.00 – Inf	0.996
cohort year [2007]	3.53	2.01 – 6.20	**<0.001**	0.09	0.01 – 0.75	**0.026**	17.68	5.55 – 56.35	**<0.001**	0.00	0.00 – Inf	1.000	0.00	0.00 – Inf	1.000	12.27	1.62 – 93.02	**0.015**	76819368.50	0.00 – Inf	0.996
cohort year [2008]	1.12	0.58 – 2.15	0.745	0.00	0.00 – Inf	0.995	0.54	0.09 – 3.22	0.496	158878004.82	0.00 – Inf	0.996	0.00	0.00 – Inf	1.000	3.65	0.43 – 31.33	0.238	34717089.91	0.00 – Inf	0.996
cohort year [2009]	7.76	4.56 – 13.20	**<0.001**	1.25	0.53 – 2.94	0.615	2.38	0.64 – 8.82	0.195	1052570849.20	0.00 – Inf	0.995	0.00	0.00 – Inf	1.000	47.00	6.50 – 340.01	**<0.001**	135079982.71	0.00 – Inf	0.996
cohort year [2010]	4.63	2.68 – 8.01	**<0.001**	0.00	0.00 – Inf	0.995	4.28	1.25 – 14.69	**0.021**	567938593.01	0.00 – Inf	0.995	0.00	0.00 – Inf	1.000	18.43	2.49 – 136.26	**0.004**	70898226.67	0.00 – Inf	0.996
cohort year [2012]	4.42	2.54 – 7.67	**<0.001**	5.58	2.64 – 11.81	**<0.001**	0.28	0.03 – 2.71	0.273	16314165.16	0.00 – Inf	0.996	0.00	0.00 – Inf	1.000	15.10	2.01 – 113.11	**0.008**	35646078.18	0.00 – Inf	0.996
cohort year [2013]	1.84	1.00 – 3.40	0.050	1.46	0.63 – 3.40	0.383	1.84	0.46 – 7.43	0.389	34169082.89	0.00 – Inf	0.996	0.89	0.05 – 14.39	0.933	9.42	1.21 – 72.99	**0.032**	9898451.86	0.00 – Inf	0.996
cohort year [2014]	0.62	0.28 – 1.36	0.231	0.00	0.00 – Inf	0.996	0.33	0.03 – 3.18	0.336	26031392.05	0.00 – Inf	0.996	0.00	0.00 – Inf	1.000	0.00	0.00 – Inf	0.994	0.94	0.00 – Inf	1.000
cohort year [2015]	1.66	0.89 – 3.08	0.111	0.00	0.00 – Inf	0.995	8.14	2.43 – 27.25	**0.001**	0.00	0.00 – Inf	1.000	0.00	0.00 – Inf	1.000	4.09	0.47 – 35.35	0.200	10887801.45	0.00 – Inf	0.996
cohort year [2016]	1.78	0.97 – 3.29	0.064	0.00	0.00 – Inf	0.995	2.89	0.78 – 10.77	0.113	0.00	0.00 – Inf	1.000	0.00	0.00 – Inf	1.000	0.00	0.00 – Inf	0.993	1.13	0.00 – Inf	1.000
cohort year [2018]	0.50	0.22 – 1.11	0.087	0.00	0.00 – Inf	0.995	0.75	0.15 – 3.74	0.730	0.01	0.00 – Inf	1.000	0.00	0.00 – Inf	1.000	0.00	0.00 – Inf	0.993	0.84	0.00 – Inf	1.000
cohort year [2019]	16.04	9.54 – 26.96	**<0.001**	0.00	0.00 – Inf	0.995	69.78	22.28 – 218.53	**<0.001**	0.02	0.00 – Inf	1.000	0.00	0.00 – Inf	1.000	99.96	13.92 – 717.64	**<0.001**	117811787.88	0.00 – Inf	0.996
cohort year [2020]	0.24	0.09 – 0.66	**0.006**	0.00	0.00 – Inf	0.995	0.46	0.08 – 2.73	0.390	0.01	0.00 – Inf	1.000	0.00	0.00 – Inf	1.000	0.00	0.00 – Inf	0.993	0.77	0.00 – Inf	1.000
cohort year [2022]	16.49	9.75 – 27.88	**<0.001**	8.28	3.90 – 17.55	**<0.001**	1.99	0.52 – 7.59	0.313	409982817.97	0.00 – Inf	0.995	88.54	12.01 – 652.74	**<0.001**	38.61	5.29 – 281.70	**<0.001**	23511662.88	0.00 – Inf	0.996
**Random Effects**																					
σ^2	NA			NA			NA			NA			7.59			NA			NA		
τ_{00}	$0.00_{case.ids}$			$0.00_{case.ids}$			$0.00_{case.ids}$			$0.00_{case.ids}$			$0.00_{case.ids}$			$0.00_{case.ids}$			$0.00_{case.ids}$		
N	$9165_{case.ids}$			$9165_{case.ids}$			$9165_{case.ids}$			$9165_{case.ids}$			$9165_{case.ids}$			$9165_{case.ids}$			$9165_{case.ids}$		
Observations	66842			67438			67438			67438			67438			67438			67438		
Marginal R^2 / Conditional R^2	NA			NA			NA			NA			1.000 / NA			NA			NA		

Table S5. Estimated risk of symptomatic DENV infection by pre-existing DENV iELISA titers, 2022-2023.

	**DENV Case**			**DENV1 Case**			**DENV3 Case**			**DENV4 Case**			**DwWS/SD**		
Predictors	*Risk Ratios*	*CI*	*p*	*Risk Ratios*	*CI*	*p*	*Risk Ratios*	*CI*	*p*	*Risk Ratios*	*CI*	*p*	*Risk Ratios*	*CI*	*p*
(Intercept)	0.06	0.04 – 0.08	**<0.001**	0.02	0.01 – 0.04	**<0.001**	0.01	0.00 – 0.02	**<0.001**	0.01	0.01 – 0.02	**<0.001**	0.00	0.00 – 0.01	**<0.001**
gm titers cat [<21]	1.62	1.21 – 2.17	**0.001**	1.10	0.65 – 1.82	0.714	2.91	1.39 – 6.09	**0.005**	2.76	1.55 – 4.96	**0.001**	2.16	1.01 – 4.61	**0.047**
gm.titers.cat21-80	1.64	1.12 – 2.34	**0.009**	1.27	0.66 – 2.32	0.459	1.90	0.69 – 5.26	0.215	3.59	1.84 – 6.89	**<0.001**	1.67	0.59 – 4.23	0.305
gm.titers.cat81-320	1.39	0.92 – 2.06	0.106	0.79	0.35 – 1.61	0.542	0.68	0.15 – 3.21	0.629	4.22	2.18 – 8.11	**<0.001**	1.80	0.62 – 4.71	0.254
gm.titers.cat321-1280	1.10	0.69 – 1.70	0.670	0.72	0.31 – 1.54	0.430	1.08	0.28 – 4.18	0.906	3.07	1.46 – 6.28	**0.003**	2.26	0.82 – 5.79	0.102
gm titers cat [>1280]	0.68	0.41 – 1.11	0.134	0.38	0.14 – 0.91	**0.041**	0.00	0.00 – Inf	0.984	2.08	0.94 – 4.49	0.067	0.69	0.17 – 2.32	0.572
age	1.04	1.00 – 1.07	**0.040**	1.05	0.99 – 1.12	0.113	1.00	0.90 – 1.10	0.961	1.05	0.99 – 1.11	0.084	1.10	1.01 – 1.19	**0.032**
sex [M]	0.91	0.74 – 1.10	0.326	1.21	0.86 – 1.71	0.272	1.10	0.64 – 1.91	0.719	0.66	0.46 – 0.93	**0.020**	0.62	0.37 – 1.01	0.060
time per imm class	1.01	0.97 – 1.06	0.614	0.99	0.92 – 1.07	0.832	1.09	0.97 – 1.23	0.165	1.01	0.93 – 1.08	0.891	1.05	0.94 – 1.16	0.378
Observations	3499			3499			3499			3499			3499		
R^2 Nagelkerke	0.027			0.015			0.056			0.059			0.048		

Table S6. Estimated risk of symptomatic DENV infection by pre-existing DENV iELISA titers, 2004-2023.

	DENV Case			DENV1 Case			DENV2 Case			DENV3 Case			DENV4 Case			DwWS/SD		
Predictors	*Risk Ratios*	*CI*	*p*	*Risk Ratios*	*CI*	*p*	*Incidence Rate Ratios*	*CI*	*p*	*Risk Ratios*	*CI*	*p*	*Incidence Rate Ratios*	*CI*	*p*	*Risk Ratios*	*CI*	*p*
(Intercept)	0.00	0.00 – 0.01	**<0.001**	0.00	0.00 – 0.00	**<0.001**	0.00	0.00 – 0.00	**<0.001**	0.00	0.00 – Inf	0.994	0.00	0.00 – 0.00	**<0.001**	0.00	0.00 – 0.00	**<0.001**
gm titers cat [<21]	1.37	1.15 – 1.63	**<0.001**	0.89	0.61 – 1.31	0.560	1.71	1.19 – 2.45	**0.003**	1.67	1.16 – 2.42	**0.006**	2.90	1.61 – 5.22	**<0.001**	1.83	1.27 – 2.64	**0.001**
gm.titers.cat21-80	1.62	1.37 – 1.92	**<0.001**	0.95	0.64 – 1.40	0.796	3.32	2.47 – 4.48	**<0.001**	1.22	0.84 – 1.77	0.286	3.59	1.83 – 7.03	**<0.001**	2.97	2.15 – 4.10	**<0.001**
gm.titers.cat81-320	1.33	1.12 – 1.58	**0.001**	0.62	0.41 – 0.95	**0.028**	2.71	2.01 – 3.67	**<0.001**	0.94	0.64 – 1.38	0.767	4.45	2.29 – 8.65	**<0.001**	2.34	1.67 – 3.27	**<0.001**
gm.titers.cat321-1280	0.96	0.77 – 1.20	0.721	0.35	0.19 – 0.63	**<0.001**	2.24	1.57 – 3.19	**<0.001**	0.51	0.29 – 0.90	**0.019**	3.14	1.50 – 6.58	**0.002**	1.68	1.12 – 2.53	**0.013**
gm titers cat [>1280]	0.64	0.48 – 0.84	**0.002**	0.25	0.12 – 0.52	**<0.001**	0.99	0.59 – 1.66	0.958	0.44	0.20 – 0.94	**0.034**	2.13	0.96 – 4.71	0.062	1.02	0.59 – 1.76	0.932
age	1.06	1.04 – 1.08	**<0.001**	1.11	1.06 – 1.17	**<0.001**	1.08	1.04 – 1.11	**<0.001**	1.01	0.96 – 1.06	0.672	1.05	0.99 – 1.11	0.078	1.08	1.05 – 1.12	**<0.001**
sex [M]	0.88	0.79 – 0.98	**0.015**	0.94	0.75 – 1.19	0.620	1.01	0.84 – 1.21	0.951	0.88	0.70 – 1.10	0.262	0.68	0.48 – 0.97	**0.034**	0.81	0.66 – 0.99	**0.039**
time per imm class	1.00	0.98 – 1.03	0.759	1.00	0.94 – 1.05	0.892	0.98	0.94 – 1.03	0.483	1.06	1.00 – 1.13	0.071	1.01	0.94 – 1.09	0.757	1.01	0.96 – 1.06	0.773
cohort year [2005]	3.50	1.99 – 6.14	**<0.001**	2.61	1.18 – 5.77	**0.018**	7.80	2.37 – 25.74	**0.001**	0.00	0.00 – Inf	1.000	0.00	0.00 – Inf	0.999	1.69	0.15 – 18.65	0.668
cohort year [2006]	0.70	0.34 – 1.48	0.354	0.10	0.01 – 0.76	**0.027**	2.47	0.67 – 9.13	0.176	0.00	0.00 – Inf	1.000	0.00	0.00 – Inf	1.000	0.78	0.05 – 12.48	0.861
cohort year [2007]	3.40	1.94 – 5.96	**<0.001**	0.09	0.01 – 0.72	**0.023**	17.01	5.31 – 54.45	**<0.001**	0.00	0.00 – Inf	1.000	0.00	0.00 – Inf	1.000	11.85	1.56 – 89.82	**0.017**
cohort year [2008]	1.05	0.55 – 2.03	0.876	0.00	0.00 – Inf	0.995	0.51	0.09 – 3.09	0.467	136901815.68	0.00 – Inf	0.996	0.00	0.00 – Inf	0.999	3.49	0.41 – 29.96	0.255
cohort year [2009]	7.28	4.28 – 12.38	**<0.001**	1.16	0.49 – 2.74	0.731	2.19	0.59 – 8.16	0.242	915708882.92	0.00 – Inf	0.995	0.00	0.00 – Inf	1.000	43.85	6.06 – 317.38	**<0.001**
cohort year [2010]	4.36	2.52 – 7.55	**<0.001**	0.00	0.00 – Inf	0.995	4.00	1.16 – 13.81	**0.028**	486842043.10	0.00 – Inf	0.995	0.00	0.00 – Inf	0.999	17.43	2.36 – 128.92	**0.005**
cohort year [2011]	1.36	0.72 – 2.57	0.346	0.31	0.09 – 1.03	0.055	0.00	0.00 – Inf	0.999	128772175.51	0.00 – Inf	0.996	0.00	0.00 – Inf	1.000	3.70	0.43 – 31.86	0.234
cohort year [2012]	4.04	2.33 – 7.02	**<0.001**	5.02	2.37 – 10.65	**<0.001**	0.25	0.03 – 2.47	0.238	13462059.59	0.00 – Inf	0.996	0.00	0.00 – Inf	0.999	13.74	1.83 – 103.04	**0.011**
cohort year [2013]	1.71	0.93 – 3.16	0.085	1.34	0.57 – 3.13	0.498	1.69	0.42 – 6.85	0.461	28828392.88	0.00 – Inf	0.996	0.87	0.05 – 14.15	0.923	8.69	1.12 – 67.38	**0.039**
cohort year [2014]	0.58	0.26 – 1.27	0.172	0.00	0.00 – Inf	0.996	0.30	0.03 – 2.91	0.299	22172025.15	0.00 – Inf	0.996	0.00	0.00 – Inf	1.000	0.00	0.00 – Inf	0.999
cohort year [2015]	1.56	0.84 – 2.91	0.160	0.00	0.00 – Inf	0.995	7.59	2.26 – 25.53	**0.001**	0.00	0.00 – Inf	1.000	0.00	0.00 – Inf	1.000	3.84	0.44 – 33.17	0.221
cohort year [2016]	1.70	0.92 – 3.14	0.088	0.00	0.00 – Inf	0.995	2.83	0.76 – 10.61	0.122	0.00	0.00 – Inf	1.000	0.00	0.00 – Inf	0.999	0.00	0.00 – Inf	0.999
cohort year [2017]	0.19	0.06 – 0.56	**0.003**	0.00	0.00 – Inf	0.995	0.00	0.00 – Inf	0.999	0.00	0.00 – Inf	1.000	0.00	0.00 – Inf	0.999	0.00	0.00 – Inf	0.999
cohort year [2018]	0.46	0.20 – 1.02	0.055	0.00	0.00 – Inf	0.995	0.75	0.15 – 3.76	0.732	0.00	0.00 – Inf	1.000	0.00	0.00 – Inf	0.999	0.00	0.00 – Inf	0.999
cohort year [2019]	14.44	8.58 – 24.31	**<0.001**	0.00	0.00 – Inf	0.995	64.24	20.40 – 202.35	**<0.001**	0.02	0.00 – Inf	1.000	0.00	0.00 – Inf	0.999	91.08	12.67 – 654.66	**<0.001**
cohort year [2020]	0.23	0.08 – 0.64	**0.005**	0.00	0.00 – Inf	0.995	0.47	0.08 – 2.85	0.414	0.00	0.00 – Inf	1.000	0.00	0.00 – Inf	1.000	0.00	0.00 – Inf	1.000
cohort year [2021]	0.14	0.04 – 0.49	**0.002**	0.11	0.02 – 0.55	**0.007**	0.00	0.00 – Inf	0.999	0.02	0.00 – Inf	1.000	0.00	0.00 – Inf	1.000	0.00	0.00 – Inf	0.999
cohort year [2022]	15.37	9.07 – 26.04	**<0.001**	7.42	3.48 – 15.81	**<0.001**	1.97	0.51 – 7.57	0.322	317888094.00	0.00 – Inf	0.995	93.98	12.72 – 694.26	**<0.001**	37.86	5.18 – 276.75	**<0.001**
**Random Effects**																		
σ^2	NA			NA			6.32			NA			7.59			NA		
τ_{00}	$0.00_{\text{case.ids}}$			$0.00_{\text{case.ids}}$			$0.00_{\text{case.ids}}$			$0.00_{\text{case.ids}}$			$0.00_{\text{case.ids}}$			$0.00_{\text{case.ids}}$		
ICC							0.00						0.00					
N	$9164_{\text{case.ids}}$			$9164_{\text{case.ids}}$			$9164_{\text{case.ids}}$			$9164_{\text{case.ids}}$			$9164_{\text{case.ids}}$			$9164_{\text{case.ids}}$		
Observations	67030			67638			67638			67638			67638			67638		
Marginal R^2 / Conditional R^2	NA			NA			0.915 / 0.915			NA			0.899 / 0.899			NA		

Table S7. Half-lives of antibody titers for a given infection history, 2004-2023.

Infection History	Average titer magnitude at 1 year post last infection*	Slope(s)**	95% Confidence Intervals	Half-lives ***	Inflection Point(s)****
DENV Immune	2.48[2.44,2.52]	**-0.12**<br>0.04	**(-0.12, -0.11)**<br>(-0.002, 0.07)	5.78<br>-17.33	8.79
DENV	1.87[1.83,1.92]	0.02<br>**-0.05**	(-0.04, 0.08)<br>**(-0.06, -0.03)**	-34.66<br>13.86	2.3
ZIKV	0.85[0.81,0.89]	**0.30**<br>**-0.19**	**(0.28, 0.33)**<br>**(-0.23, -0.16)**	-2.31<br>3.65	3.2
DENV-DENV	2.71[2.61,2.80]	**-0.40**<br>**-0.04**	**(-0.55, -0.26)**<br>**(-0.16, -0.02)**	1.73<br>17.33	2.31
DENV-ZIKV	3.01[2.98,3.13]	**-0.08**<br>-0.18	**(-0.11, -0.05)**<br>(-0.89, 0.53)	8.66<br>3.85	5
ZIKV-DENV	3.34[3.25,3.44]	**-0.28**	**(-0.35, -0.21)**	2.48	None
2+DENV	2.96[2.90,3.01]	**-0.36**<br>**-0.03**	**(-0.44, -0.29)**<br>**(-0.05, -0.02)**	1.93<br>23.10	2.3
2+DENV-ZIKV	3.56[3.44,3.67]	**-0.41**<br>0.16<br>-0.23	**(-0.58, -0.23)**<br>(-0.05, 0.37)<br>(-0.49, 0.03)	1.69<br>-4.33<br>3.01	2.4<br>4.4

*Predicted log10(DENV iELISA titer) and confidence intervals at 1 year after last infection.
** log10(DENV iELISA titer)/year. Significant slopes are bolded.
***Half-lives were calculated by computing log(2) divided by slope (log(2)/slope). Negative half-lives indicate an increase in titer magnitude.
**** Inflection points were calculated in years
